# Efficacy of oral metronomic chemotherapy in the management of head and neck squamous cell carcinoma—a systematic review

**DOI:** 10.3389/froh.2025.1632316

**Published:** 2025-07-30

**Authors:** Aardra Binithadas Segin Chandran, Deepak Pandiar, Reshma Poothakulath Krishnan, Divya Gopinath

**Affiliations:** ^1^ Department of Oral Pathology and Microbiology, Saveetha Dental College and Hospitals, Saveetha Institute of Medical and Technical Sciences, Saveetha University, Chennai, India; ^2^ College of Dentistry, Ajman University, Ajman, United Arab Emirates; ^3^ Centre of Medical and Bio-Allied Health Sciences Research, Ajman University, Ajman, United Arab Emirates

**Keywords:** oral metronomic chemotherapy, head and neck squamous cell carcinoma, survival rate, angiogenesis, OSCC (oral squamous cell carcinoma)

## Abstract

**Background:**

Head and neck squamous cell carcinoma (HNSCC) is a group of heterogeneous malignancies and constitutes one of the most prevalent forms of cancer. Oral metronomic chemotherapy (OMCT) is a treatment in which low doses of anticancer drugs are given at regular intervals over a long time, with many advantages over conventional therapies, particularly in nations with high cancer burden. The present systematic review aimed to evaluate the efficacy of OMCT in the management of HNSCC in comparison to other standard chemotherapy regimens. Methodology: The review was registered in the Prospero database (CRD42023426000). An electronic search was conducted using PubMed, Scopus, Web of Science and Google Scholar databases. Articles in which OMCT was used to treat HNSCC were included for systematic review, and the survival and response rates were analyzed.

**Results:**

Twenty-four eligible articles were included for evaluation, which revealed that administration of OMCT produced higher survival and response rates in subjects compared to standard chemotherapy.

**Conclusion:**

The evidence from the included studies supports that oral metronomic chemotherapy is substantially more effective as compared to standard chemotherapy regimens in squamous cell carcinomas of the head and neck.

**Systematic Review Registration:**

https://www.crd.york.ac.uk/PROSPERO/view/CRD42023426000, identifier (CRD42023426000).

## Introduction

Head and neck squamous cell carcinoma (HNSCC) is ranked as the seventh most common cancer in the world and has been frequently linked to tobacco and alcohol use, as well as human papillomavirus (HPV), *Helicobacter pylori* and *Candida albicans* infections (1–3). In cases of recurrent or metastatic cancer, conventional therapeutic approaches such as surgery, radiation and systemic chemotherapy frequently result in considerable morbidity and have poor efficacy (4, 5). Oral metronomic chemotherapy (OMCT) has become a viable option due to its ability to suppress tumor angiogenesis, modulate the immune system, and minimize toxicity (6). It entails frequently administering low-dose chemotherapy drugs without extended intervals.

Angiogenesis is a process by which new blood vessels proliferate to deliver nourishment to the tumor cells (7). Additionally, alternate mechanisms such as vasculogenic mimicry have been described in various human malignancies (8). Inhibiting angiogenesis is the main mechanism by which metronomic chemotherapy functions (9). The anti-angiogenic action of metronomic chemotherapy relies upon targeting the endothelial cells in the tumor vasculature, which are more susceptible to continual low-dose chemotherapy. Furthermore, it is believed that metronomic chemotherapy has immunomodulatory effects, specifically through the reduction of regulatory T-cells (Tregs), which are responsible for inhibiting the body's immune response against tumors (6). These processes differ from the classic cytotoxic effects of chemotherapy, which are designed to destroy rapidly dividing tumor cells.

Oral metronomic chemotherapy has been researched in several therapeutic contexts, especially for patients with recurrent or metastatic HNSCC who have few alternative options for treatment. OMCT could be a better alternative for patients who are not promising candidates for aggressive therapy, according to a phase II study assessing the use of low-dose oral cyclophosphamide in combination with celecoxib in patients with advanced HNSCC (10). The study found that a subset of patients experienced a significant reduction in tumor size with minimal toxicity. Patients receiving OMCT also experienced fewer adverse effects, such as neutropenia and mucositis, which are common in standard chemotherapy (11).

OMCT has also been explored as a maintenance therapy to prolong disease control following induction chemotherapy or chemoradiation (12). A study reported that patients with HNSCC who received maintenance OMCT with methotrexate and celecoxib had a longer median PFS compared to those who received no further treatment after completing standard therapy (13). This suggests that OMCT may help in sustaining the therapeutic response and delaying disease progression. An area of growing attention is the use of immune checkpoint inhibitors in conjunction with OMCT. Metronomic chemotherapy has been demonstrated in preclinical investigations to increase the effectiveness of immunotherapy by altering the tumor microenvironment and promoting the infiltration of cytotoxic T-cells (1). Many clinical trials are now underway to assess the synergistic effects of OMCT in HNSCC (14).

The present systematic review was thus designed to evaluate the efficacy of oral metronomic chemotherapy in the management of head and neck squamous cell carcinoma in comparison to other standard chemotherapy regimens.

## Methodology

### Protocol and registration

The Preferred Reporting Items for Systematic Reviews and Meta-Analyses [PRISMA (15)] guidelines were used to design this systematic review (9). This review was registered at the International Prospective Register of Systematic Reviews database (CRD42023426000). The research question was, “Does oral metronomic chemotherapy play any role in the treatment of head and neck squamous cell carcinoma?” The PICO for the present systematic review was as follows:

• Population: Head and neck squamous cell carcinoma, including all the subsites
• Intervention: Oral metronomic chemotherapy or low-dose chemotherapy
• Control: Conventional treatment, including surgical intervention with or without adjuvant therapies
• Outcome: Comparison of the efficacy of OMCT with conventional therapy

### Eligibility criteria

All relevant articles obtained from information sources (PubMed, SCOPUS, Web of Science and Google Scholar) were screened and included in the review only if the papers were original research studies, full-length text was available irrespective of the language, the studies were conducted only on human participants, and the studies included an analysis of oral metronomic chemotherapy in head and neck squamous cell carcinoma.

### Information sources and search strategy

Two authors (ABSC and DP) independently searched PubMed, SCOPUS, Web of Science and Google Scholar for the keywords alone and in combination, followed by a manual search and assessment of cross references. The MeSH terminology formulated for each database for the literature search was (TITLE-ABS-KEY (metronomic AND chemotherapy) AND TITLE-ABS-KEY (head AND neck AND squamous AND cell AND carcinoma)).

### Selection and data collection process

The same authors individually screened the titles and abstracts of all the articles. The papers that did not meet the eligibility criteria were excluded. Full-length texts were downloaded for all the eligible articles. The complete articles were read and evaluated for eligibility, and the reasons for exclusion were recorded. The third author (RPK) was involved in resolving the discordance if any discrepancy was noted. The following information was extracted from each included article: author(s), country of origin, year of publication, study design, number of cases, number of controls, drug or drug combination used for oral metronomic chemotherapy, and control treatment. The criteria for evaluation the efficacy of OMCT were divided into two main categories: (1) primary study end point (Overall survival) and (2) secondary end points. The secondary end points included progression-free survival (PFS), disease-free survival (DFS), distant metastasis-free survival (DMFS), disease-specific survival (DSS), complete response (CR), partial response (PR), stable disease (SD), progressive disease (PD), and clinical benefit rate (CR + PR + SD). For the assessment of overall survival, death due to any cause was considered as the event, while distant metastasis, death from any cause, were considered as events in the secondary end points. Any response other than disease progression was regarded as clinical response. All included studies were carefully assessed for evaluation and recording of the end points post-OMCT.

### Statistical analysis

The quantitative data were tabulated and processed in Microsoft Excel 2021 (Microsoft Corporation, Redmond, Washington, United States) and analyzed descriptively. For estimating means, the data were processed using IBM SPSS Statistics for Windows, Version 26.0 (Released 2019; IBM Corp., Armonk, New York, United States), and means and standard deviations were estimated.

### Risk of bias analysis

The revised Cochrane risk-of-bias tool for randomized trials (RoB 2) was used to assess the risk of bias for randomized controlled trials (16), and the Newcastle Ottawa scale was employed for the included cross-sectional and case-control studies (17). There are five domains in RoB 2 tool, including Domain 1: Risk of bias arising from the randomization process, Domain 2: Risk of bias due to deviations from the intended interventions (effect of assignment to intervention), Domain 3: Missing outcome data, Domain 4: Risk of bias in the measurement of the outcome and Domain 5: Risk of bias in the selection of the reported result. Each domain contains answerable questions, where the answer is provided as “yes”, “no”, “partial yes”, “partial no” or “not included”. The entire risk based on the domains is expressed as “low risk”, “high risk” or “some concerns”.

The Newcastle Ottawa scale for cross-sectional and case-control studies bears slight differences. Regarding cross-sectional studies, the three categories include selection, exposure, and comparability. For selection and exposure, a maximum of one star is assigned for each numbered parameter for each included study. While assessing comparability, a maximum of two stars could be given. Each category was judged by two authors (DP & RPK), and any discordance was resolved by discussion with the third observer (ABS). Studies that obtained ≥7 or more stars, 4–6 stars and ≤3 stars were respectively marked as having “low risk”, “high risk” and “very high” risk of bias. The scale is similar for case-control studies with slight modifications, where the third category is exposure instead of outcome.

## Results

**Figure 1:**
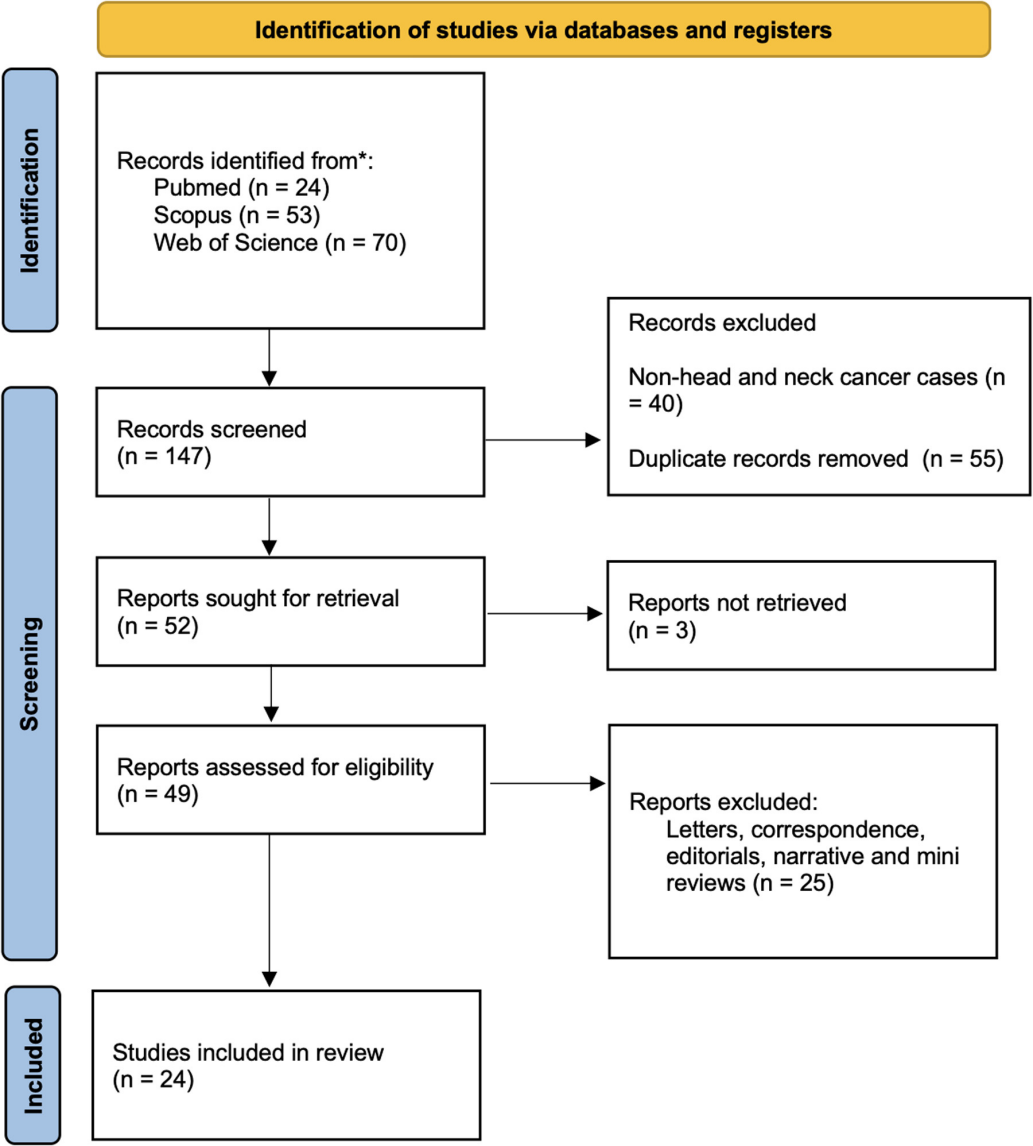
Flowchart showing the article selection process according to the preferred reporting items for systematic reviews and meta-analyses (PRISMA) 2020 guidelines; *deciphers reporting the number of records identified from each database or register searched (no records were found from other sources).

The literature search strategy revealed 147 articles published until 2024 in various electronic scientific databases. Out of these 147 articles, forty papers were excluded after reading the titles and abstracts for eligibility, along with 55 duplicate papers, yielding 52 articles for inclusion in the review. These 52 articles were further evaluated by reading the full text for eligibility. At this stage, three articles were excluded as the full texts could not be retrieved. From the 49 full-text articles that were retrieved, 25 articles were again excluded based on the eligibility criteria. Finally, 24 articles were included in the present systematic review (Figure 1) (18–41).

### Characteristics of the included studies

All the included studies were published between 2009 and 2024. Out of the 24 included studies, 16 were from India (19–21, 23, 25, 28, 30, 32, 35, 38, 40), four studies were from Japan (24, 34, 39, 41),, two from Taiwan (29, 33), one from Spain (18) and one from the USA (22) (Table 1). A total of 1964 OMCT cases and 1,266 control cases were included. There were six randomized controlled trials (RCTs) (21, 22, 30, 32, 38, 40), nine cross-sectional studies (18, 19, 26, 28, 31, 35, 36, 37, 39), and nine case-control studies (20, 23–25, 27, 29, 33, 34, 41). The data regarding the measures of outcome were heterogeneous. While most studies analyzed the overall survival of the patients, others studied parameters, included progression-free survival, disease-free survival, distant metastasis-free survival, disease-specific survival, complete response/partial response, stable disease/progressive disease, and the clinical benefit rate. There were nineteen studies where OS was analyzed (19, 21–25, 27–35, 38–40), nine studies analyzed progression-free survival (PFS) (19, 21, 22, 26, 27, 35, 40), six studies evaluated DFS (20, 23, 24, 29, 33, 41), DMFS was reported in three studies (24, 33, 34), another study analyzed disease-specific survival (29), eight studies analyzed both OS and PFS (19, 21, 22, 27, 30, 31, 35, 40), and five studies analyzed both OS and DFS (23, 24, 29, 33, 41). Regarding a control group for comparison, no controls were included in 11/24 studies (18, 19, 25, 26, 28, 30, 31, 35–37, 39). Conventional surgical approaches with or without adjuvant therapy were instituted in the studies with controls.

**Table 1:** Clinico-demographic details of the twenty-four included studies in the present systematic review.

Sl No	Author	Year	Country	Type of study	Cases	Controls	Drug delivered with dose	Control with dose	Interpretation for cases	Interpretation for controls
1	Grau et al. (18)	2009	Spain	Cross-sectional	60	–	Paclitaxel 80 mg/m^2^ weekly	Nil	CR 0% + PR 43.3% + SD 15% + PD 38.3%	–
2	Patil et al. (19)	2012	India	Cross-sectional	18	–	Celecoxib 200 mg twice daily + Methotrexate 15 mg/m^2^ daily	Nil	PFS 5 months + OS 3.05 months	–
3	Pai et al. (20)	2013	India	Case-control	32	32	Methotrexate 15 mg/m^2^ weekly + Celecoxib 200 mg twice daily	Nil	2 year DFS 86.5%	2 year DFS 71.6%
4	Patil et al. (21)	2015	India	Randomized controlled trial	57	52	Celecoxib 200 mg twice daily + methotrexate 15 mg/m^2^ weekly	Cisplatin 75 mg/m^2^ 3-weekly	PFS 101 days + OS 249 days	PFS 66 days + OS 152 days
5	Swiecicki et al. (22)	2016	USA	Randomized controlled trial	11	24	Docetaxel + Metronomic AT-101	Docetaxel alone Docetaxel + Pulse dose AT-101	PFS 4.2 months + OS 5 months + CR 0% + PR 18% + SD 55% + PD 27%	PFS 4.5 months + OS 8.3 months + CR 0% + PR 8% + SD 77% + PD 0%|PFS 2.8 months + OS 4.9 months + CR 0% + PR 9% + SD 55% + PD 27%
6	Pandey et al. (23)	2016	India	Case-control	130	205	Methotrexate 15 mg/m^2^ weekly + Celecoxib 200 mg twice daily	Nil	DFS 14 months + OS 26 months	DFS 8 months + OS 14 months
7	Kina et al. (24)	2016	Japan	Case-control	63	54	Bleomycin 15 mg over 1hr twice weekly for 3 weeks + 450 mg UFT-E granules thrice daily/S-1 granules 100 mg daily	Nil	OS 90% + DMFS 90% + 5 year DFS 63%	OS 76% + DMFS 76% + 5 year DFS 55%
8	Patil et al. (25)	2016	India	Case-control	5	3	Methotrexate 15 mg/m^2^ weekly + Celecoxib 200 mg twice daily	Nil	OS 126 days	–
9	Patil et al. (26)	2016	India	Cross-sectional	15	–	Methotrexate 15 mg/m^2^ weekly + celecoxib 200 mg twice daily + erlotinib 150 mg once daily	Nil	PFS 148 days	–
10	Patil et al. (27)	2017	India	Case-control	60	60	Methotrexate 15 mg/m^2^ weekly + Celecoxib 200 mg twice daily	Paclitaxel 80 mg/m^2^ + Cetuximab (loading dose 400 mg/m^2^ followed by 250 mg/m^2^) weekly	Clinical benefit rate 36.7% + PFS 101 days + OS 191 days	Clinical benefit rate 75% + PFS 173 days + OS 256 days
11	Patil et al. (28)	2017	India	Cross-sectional	340	–	Methotrexate 15 mg/m^2^ weekly + Celecoxib 200 mg twice daily	Nil	OS 150 days	–
12	Hsieh et al. (29)	2018	Taiwan	Case-control	114	242	Tegafur-uracil 100−400 mg daily	Nil	5 year DFS 57% + 5 year DSS 74% + 5 year OS 65%	5 year DFS 41% + 5 year DSS 61% + 5 year OS 48%
13	Patil et al. (30)	2019	India	Randomized controlled trial	91	–	Erlotinib 150 mg once daily + celecoxib 200 mg twice daily + methotrexate 3–15 mg/m^2^ weekly	Nil	PFS 4.6 months + OS 7.17 months	–
14	Harsh et al. (31)	2020	India	Cross-sectional	84	–	Celecoxib 200 mg twice daily + methotrexate 15 mg/m^2^ weekly	NIl	PFS 110 days + OS 195 days	–
15	Patil et al. (32)	2020	India	Randomized controlled trial	213	209	Methotrexate 15 mg/m^2^ weekly + celecoxib 200 mg twice daily	Cisplatin 75 mg/m^2^ 3-weekly	OS 7.5 months	OS 6.1 months
16	Yeh et al. (33)	2021	Taiwan	Case-control	96	144	Tegafur-uracil 100–400 mg daily	Nil	DFS 54.5 months + OS not reached + DMFS not reached	DFS 54.5 months + OS 54.1 months + DMFS not reached
17	Kina et al. (34)	2021	Japan	Case-control	70	45	Bleomycin with UFT-E (59%) + Bleomycin with S-1 (41%)	Nil	DMFS 84.8% + OS 84.8%	DMFS 75.6% + OS 75.6%
18	Kashyap et al. (35)	2021	India	Cross-sectional	14	–	NACT—paclitaxel 175 mg/m^2^ + carboplatin every 3 weeks|OMCT—methotrexate 9 mg/m^2^ weekly + celecoxib 200 mg twice daily + erlotinib 150 mg once daily	Nil	PFS 11.4 months + OS not reached	–
19	Sultania et al. (36)	2021	India	Cross-sectional	134	–	Methotrexate 15 mg/m^2^ weekly + Celecoxib 200 mg twice daily	Nil	CR 2.5% + PR 46.6% + SD 39.8% + DP 11%	–
20	Shenoy et al. (37)	2022	India	Cross-sectional	68	–	Methotrexate 15 mg/m^2^ weekly + celecoxib 200 mg twice daily + erlotinib 100 mg once daily	Nil	PR 54% + SD 34% + PD 3%	–
21	Patil et al. (38)	2023	India	Randomized controlled trial	76	75	Methotrexate 9 mg/m^2^ weekly + celecoxib 200 mg twice daily + erlotinib 150 mg once daily + nivolumab 20 mg 3-weekly	Methotrexate 9 mg/m^2^ weekly + celecoxib 200 mg twice daily + erlotinib 150 mg daily	OS 10.1 months	OS 6.7 months
22	Kina S at al (39)	2023	Japan	Cross-sectional	40	–	S-1 100 mg daily + Bleomycin 15 mg bolus	Nil	OS 98%	OS 82%
23	Patil et al. (40)	2023	India	Randomized controlled trial	68	69	Methotrexate 15 mg/m^2^ weekly + celecoxib 200 mg twice daily	Nil	PFS 60.8% + OS 62.4%	PFS 68.7% + OS 79.4%
24	Kina et al. (41)	2024	Japan	Case-control	106	54	S-1 120 mg twice daily for 2 weeks	Nil	5 year OS 96% + 5 year DFS 91%	5 year OS 81% + 5 year DFS 70%

OS, overall survival; PFS, progression-free survival; DFS, disease-free survival; DMFS, distant metastasis-free survival; DSS, disease-specific survival; CR-complete response; PR, partial response; SD, stable disease; PD, progressive disease; CR + PR + SD, clinical benefit rate.

### Demographic data

The demographic data of the case and control patients were retrieved from all 24 studies. Out of the 1964 case and 1,269 control participants, 2,685 (83%) participants were male and 548 (17%) were female (M:F ratio of 4.89:1).

### Overall survival (OS)

As aforementioned, nineteen (19/24; 79.2%) of the twenty-four studies examined the overall survival (OS) to assess the effectiveness of OMCT. Yet again, there was geneity in the presentation of values. 13/19 (68.4%) studies reported the length (in months) of OS for the participants (19, 21–23, 26–28, 30–33, 35, 37), while the remaining 6 (31.6%) studies presented survival in percentage (24, 29, 34, 39–41). Twelve (70.6%) studies compared the overall survival rates in OMCT and control treatments; 9/12 (75%) studies showed that the OMCT group had higher overall survival rates (21, 23, 24, 29, 32, 34, 38, 39, 41). The mean overall survival duration was calculated, which yielded a value of 6.85 months, whereas the mean overall survival as estimated in percentage was 82.7%.

### Progression free survival (PFS)

Nine out of twenty-four studies reported the end point measurement as progression-free survival (PFS) (19, 21, 22, 26, 27, 30, 31, 35, 40). Similar to OS data, there was heterogeneity in the presentation of values. While most of the included studies presented values in months, Kina et al., showed the data values in percentages (41). Out of nine studies, 5 (55.6%) did not have any control group for comparison. In the studies, where a control group was available, it was found that the PFS was higher in control groups (22, 27, 40). The estimated mean progression-free survival duration was 5.07 months, and the mean progression-free survival percentage was 60.8%.

### Disease free survival

Out of the 24 studies, 6 (25%) evaluated the efficacy of OMCT by analyzing the disease-free survival (DFS) of the participants (20, 23, 24, 29, 33, 41). The disease-free survival was estimated for two years, three years and 5 years, respectively, in one (20), three (24, 29, 41) and two studies (23, 33). Out of the six studies, Yeh et al., showed equal duration of disease-free survival in both the OMCT and control groups (33), while the remaining five showed that the DFS was higher when OMCT was administered (20, 23, 24, 29, 41). The estimated mean disease-free survival duration was 34.25 months, and the mean disease-free survival percentage was 74.38%.

### Distant metastasis free and disease-specific survival

Out of the 24 studies, 3 (12.5%) studies (14, 23, 24) evaluated the efficacy of OMCT by analyzing the distant metastasis-free survival (DMFS) of the participants (24, 33, 34). It was shown that the patients who were treated with OMCT had longer distant metastasis-free survival rates; the mean distant metastasis-free survival percentage was 87.4%. In one of the included papers, the author estimated disease-specific survival and showed a higher disease-specific survival rate of 74% in the OMCT group compared to 61% in the control group.

### Complete or partial response

Complete response was the parameter of assessment in three studies, and the mean complete response percentage was 0.83% (18, 22, 36), while the percentage of mean partial response was 40.48%, based on the four included studies (18, 22, 36, 37).

### Progression of disease: stable disease/progressive disease

Out of the included twenty-four studies, the progression of the disease was analyzed in four papers (18, 22, 36, 37). However, only one group of authors included a control group for comparison, where the percentage of patients showing stability/progression of disease was found to be equal in the study group and controls (22). The mean stable disease and progressive disease percentages were estimated to be 35.95% and 19.83%, respectively.

### Clinical benefit rate (CR + PR + SD)

The clinical benefit rate of the participants was evaluated by Patil VM et al., and this study reported a higher clinical benefit rate of 75% in the control group compared to 36.7% in the OMCT group (27).

### Details of OMCT treatment regimens

In general, Methotrexate (9 mg/m^2^ weekly–15 mg/m^2^ weekly) was used in most studies as the primary regimen in combination with other drugs (20, 21, 23, 25–28, 32, 36, 38). In five included papers, a control group was used for comparison (21, 22, 27, 32, 38). Out of these five stuidies, in three papers, Methotrexate (9 mg/m^2^ weekly–15 mg/m^2^ weekly) was used in combination with other drugs like celecoxib, erlotinib and nivolumab, and it was found that in two of these three studies, the overall survival was better than the control group (21, 32, 38). A lower survival was noted where Paclitaxel was used in the control group (27). Other regimens used as OMCT were Docetaxel + Metronomic AT-101 (22), which was used in comparison with Docetaxel + Pulse dose AT-101 or Docetaxel alone as control. Interestingly, the survival for cases (5 months) in comparison with Docetaxel + Pulse dose AT-101 control group was similar (4.9 months), however, the Docetaxel alone group yielded a higher survival of 8.3 months. Other regimens used as OMCT were, Paclitaxel 80 mg/m^2^ weekly (18), Bleomycin (15 mg over 1hr twice weekly for 3 weeks + 450 mg UFT-E granules thrice daily/S-1 granules 100 mg daily (24), Tegafur-uracil 100–400 mg daily (29, 33), Erlotinib 150 mg once daily with celecoxib and methotrexate (30), and S-1 100 mg daily with 15 mg bolus of Bleomycin (30).

### Risk of bias analysis

Four of the six included RCTs showed a high overall risk of bias (21, 22, 30, 40), and the remaining 2 had some concerns (32, 38) (Table 2). Regarding cross-sectional studies, only two studies showed a score of seven and were categorized as having low risk (18, 36), and the other seven had a high risk of bias (19, 26, 28, 31, 35, 37, 39) (Table 3). Out of the nine case-control studies, six studies had a score of seven or more and bore a low risk (24, 27, 29, 33, 24, 41), while the remaining three studies had a high risk of bias (20, 23, 25) (Table 4).

**Table 2:** Cochrane risk-of-bias tool for randomized trials version 2 (RoB 2).

Study and year	Domain 1	Domain 2	Domain 3	Domain 4	Domain 5	Overall
Patil et al. (21)	High Risk	Some concerns	Low Risk	Low Risk	High Risk	High Risk
Swiecicki et al. (22)	High Risk	Some concerns	Some concerns	Low Risk	Low Risk	High Risk
Patil et al. (30)	High Risk	Some concerns	Low Risk	Low Risk	High Risk	High Risk
Patil et al. (32)	Low Risk	Some concerns	Low Risk	Low Risk	Low Risk	Some concerns
Patil et al. (38)	Low Risk	Some concerns	Low Risk	Low Risk	Low Risk	Some concerns
Patil et al. (40)	Low Risk	Some concerns	Low Risk	Low Risk	High Risk	High Risk

In this color-coded ranking, green color represents a low risk of bias, yellow some concerns, and red a high risk of bias.

**Table 3:** Quality assessment tool for the included cross-sectional studies using Newcastle-Ottawa scale.

S. No.	Authors and year of publication	Selection				Comparability	Outcome		Summary scores
		Representativeness of sample	Sample size	Ascertainment of the exposure	Non respondents	The subject in different outcome groups are comparable based on the study design or analysis, confounding factors are controlled	Assessment of outcome	Statistical tests	
1	Grau et al. (18)	*		**		**	*	*	7
2	Patil et al. (19)	*		**		**	*		6
3	Patil et al. (26)	*		**		**	*		6
4	Patil et al. (28)	*				**	*	*	5
5	Harsh et al. (31)	*		**		**	*		6
6	Kashyap et al. (35)			**		**	*		5
7	Sultania et al. (36)	*		**		**	*	*	7
8	Shenoy et al. (37)	*		**		**	*		6
9	Kina et al. (39)	*				**	*	*	5

**Table 4:** Quality assessment tool for the included case-control studies using Newcastle-Ottawa scale.

S. No.	Authors and year of publication	Selection				Comparability	Exposure			Summary scores
		Adequate case definition	Representativeness of the cases	Selection of controls	Definition of controls	Comparability of cases and controls on the basis of the design and analysis	Ascertainment of exposure	Same method of ascertainment for cases and controls	Non- response rate	
1	Pai et al. (20)	*				**	*	*	*	6
2	Pandey et al. (23)	*	*			*	*	*	*	6
3	Kina et al. (24)	*	*		*	**	*	*	*	8
4	Patil et al. (25)	*				**	*	*	*	6
5	Patil et al. (27)	*	*		*	**	*	*	*	8
6	Hsieh et al. (29)	*	*			**	*	*	*	7
7	Yeh et al. (33)	*	*		*	**	*	*	*	8
8	Kina et al. (34)	*	*		*	**	*	*	*	8
9	Kina et al. (41)	*	*		*	**	*	*	*	8

## Discussion

With few treatment options and a poor prognosis, head and neck squamous cell carcinoma (HNSCC) is still a serious health concern, particularly in underdeveloped and developing nations where the resources are limited, with added financial burden and diagnosis of the disease at an advanced stage (42–44). Although surgery, with adjuvant chemotherapy and radiotherapy (CTRT), remains the mainstay treatment for head and neck squamous cell carcinoma, the high dosage and repeated cycles of CTRT are usually linked to serious side effects, which makes them unsuitable for patients with advanced or recurrent disease (45, 46). This has raised interest in metronomic chemotherapy, a method of administering chemotherapeutics continuously at low doses to modify immune responses, lower angiogenesis, and target the tumor microenvironment without the significant toxicity of traditional regimens (47–49). OMCT is not only advantageous in the advanced clinical stage or recurrent diseases but could also be provided to patients where the waiting period is long or in large unresectable tumors with difficulty in obtaining free margins. This holds particularly true for the malignancies of the posterior part of the oral cavity. Preliminary studies suggest that OMCT may offer an effective and less toxic alternative for advanced HNSCC, though robust clinical evidence is still needed (18–22). The present systematic review was performed to evaluate the efficacy of OMCT in HNSCC, aiming to expand treatment options for this challenging patient population and to investigate its potential as a sustainable, less invasive therapeutic approach.

From the systematic review conducted, it was found that the studies where controls were included for comparison of the efficacy of OMCT in head and neck squamous cell carcinoma, most studies demonstrated that the overall survival with OMCT was better as compared to the control groups (20, 21, 23, 24, 29, 32, 34, 38, 39, 41). Few studies, however, showed a reverse trend (22, 27, 40). Owing to the fact that the values of end point determination were expressed variably in percentage, months, years or days, homogeneous data could not be generated to assess the significance and to perform a meta-analysis. The longest duration of overall survival was 26 months observed with the weekly administration of 15 mg/m^2^ of Methotrexate and twice-daily administration of 200 mg of Celecoxib (23). In contrast, the control group showed an overall survival of only 14 months. Similar results were obtained from other studies. The highest percentage of overall survival was 98% observed with the daily administration of 100–400 mg of Tegafur-Uracil, contrasting with the 82% OS in the control group (39). Similarly, the longest duration of progression-free survival was 11.4 months, which was observed with the combination of neoadjuvant chemotherapy (175 mg/m^2^ of Paclitaxel with Carboplatin every 3 weeks) and oral metronomic chemotherapy (9 mg/m^2^ of Methotrexate weekly with 200 mg of Celecoxib twice daily, and 150 mg of Erlotinib once daily) (35). The highest percentage of progression-free survival was 60.8% observed with the weekly administration of 15 mg/m^2^ of Methotrexate and twice-daily administration of 200 mg of Celecoxib (40). The longest duration of disease-free progression was 54.5 months observed with weekly administration of 15 mg/m^2^ of Methotrexate and twice-daily administration of 200 mg of Celecoxib (33). The highest percentage of disease-free survival was 91% observed with the twice-daily administration of 120 mg of S-1 for 2 weeks (41) (Table 1).

The highest degree of suitable results was obtained with the oral metronomic administration of the combination of Methotrexate and Celecoxib. The mechanism of action of Methotrexate is due to its ability to inhibit the enzymes responsible for nucleotide synthesis, including dihydrofolate reductase (DHFR), thymidylate synthase (TS), aminoimidazole carboxamide ribonucleotide transformylase (AICART), and amido phosphoribosyl transferase (APRT) (50). As a result, methotrexate prevents tumor cells from proliferating and also has additional anti-inflammatory effects through several mechanisms, including adenosine signaling, the generation of reactive oxygen species (ROS), the decrease of pro-inflammatory cytokine levels, and the enhancement of immune balance through the increase in Treg cells (51).

The usage of a combination of celecoxib with methotrexate was commonly administered as OMCT and showed better outcome measures, as evidenced in the present review (33). Celecoxib inhibits the 3-phosphoinositide-dependent kinase-1 (PDK-1) signaling pathway and binds to the cadherin-11 (CDH11) protein, which is believed to be involved in the development of cancers, to provide anticancer effects (52). It mainly regulates the proliferation, migration, and invasion of tumor cells by inhibiting the cyclooxygenase 2 (COX2)- prostaglandin E2 (PGE2) signal axis, thereby inhibiting the phosphorylation of nuclear factor-*κ*-gene binding and the expression of matrix metalloproteinases 2 (MMP2) and 9 (MMP9). Celecoxib also promotes the apoptosis of tumor cells by enhancing mitochondrial oxidation, thereby activating the mitochondrial apoptotic process. Celecoxib further reduces drug resistance by increasing the sensitivity of cancer cells to chemotherapy drugs (6). The above mechanisms of methotrexate and celecoxib show that these suppress tumor growth by inhibiting cell division and promoting apoptosis of tumor cells, respectively. These two mechanisms work hand in hand to provide the heightened anticancer effect.

Our compiled results are in line with earlier studies that demonstrated the possible advantages of OMCT in HNSCC (25–30). A better overall immunological response may result from the persistently low dosage, which may lessen immune cell suppression (47). Low-dose, continuous chemotherapy is given orally as part of OMCT, which makes administration simpler and increases patient compliance. According to earlier research, metronomic chemotherapy may be able to control tumor growth with fewer side effects than traditional high-dose regimens by targeting the tumor microenvironment, namely by preventing angiogenesis and regulating the immune response (48, 53).

The current review has, however, a few limitations, including a small sample size and limited follow-up, indicating the need for larger randomized trials. The reporting of survival results, such as the duration of survival in some studies and the percentage of surviving patients in other studies, makes it difficult to effectively compare the results of the studies and also hinders the conduct of a meta-analysis of the extracted data. 67% of the included studies were conducted in India, which may have affected the results, as previous research shows that pharmaco-ethnicity impacts the treatment outcomes of patients undergoing various forms of anti-cancer therapy, primarily through polymorphism within the genes responsible for metabolism (54).

Standardization of reporting criteria is the next step in the systematic analysis of studies into oral metronomic chemotherapy, as it facilitates the proper comparison of treatment outcomes and statistical analysis of the efficacy of various metronomic drug dosages and multidrug combinations. Future studies should also explore biomarkers to identify patients most likely to benefit from OMC and examine combinations with immunotherapies for enhanced efficacy (8). The evidence supporting the efficacy of OMCT in HNSCC is promising, particularly in the context of recurrent or metastatic disease, where traditional treatments often fail. The advantages of OMCT include its favorable toxicity profile, ease of administration, and potential to be combined with other therapeutic modalities, such as immunotherapy. However, challenges remain, including identifying the optimal dosing regimens, understanding the long-term effects, and determining the patient populations that would benefit the most from this approach.

## Conclusion

Based on the data collected and analyzed, oral metronomic chemotherapy showed a comparatively better survival compared to the standard chemotherapy regimens, particularly in the case of squamous cell carcinomas of the head and neck. However, it must be noted that there was heterogeneity in the usage of drug regimen and dosages among various studies, necessitating large scale multicentric studies for affirmation of the findings. The anti-angiogenic and immunomodulatory effects of OMCT, combined with its low toxicity, make it an attractive alternative to conventional chemotherapy. The utilization of OMCT for anti-cancer treatment has the added advantage of producing decreased drug resistance, which thereby increases the efficacy of the administered treatment.

## Data Availability

The original contributions presented in the study are included in the article/Supplementary Material, further inquiries can be directed to the corresponding authors.
